# Diagnostic Test Efficacy of Meibomian Gland Morphology and Function

**DOI:** 10.1038/s41598-019-54013-4

**Published:** 2019-11-22

**Authors:** Jiaxin Xiao, Muhammed Yasin Adil, Jonatan Olafsson, Xiangjun Chen, Øygunn A. Utheim, Sten Ræder, Neil S. Lagali, Darlene A. Dartt, Tor P. Utheim

**Affiliations:** 1 Department of Medical Biochemistry, Oslo University Hospital, Oslo, Norway; 2 Institute of Clinical Medicine, Faculty of Medicine, University of Oslo, Oslo, Norway; 3 The Norwegian Dry Eye Clinic, Oslo, Norway; 4 Department of Ophthalmology, Sørlandet Hospital, Arendal, Norway; 5 Department of Ophthalmology, Oslo University Hospital, Oslo, Norway; 6 Department of Plastic and Reconstructive Surgery, Oslo University Hospital, Oslo, Norway; 7 Department of Ophthalmology, Department of Clinical and Experimental Medicine, Linköping University, Linköping, Sweden; 8 Schepens Eye Research Institute, Massachusetts Eye and Ear Infirmary, Department of Ophthalmology, Harvard Medical School, Boston, Massachusetts United States; 9 Institute of clinical dentistry/Department of oral biology, Faculty of Dentistry, University of Oslo, Oslo, Norway; 10 Faculty of Health and Social Sciences, Department of Optometry, Radiography and Lighting Design, National Centre for Optics, Vision and Eye Care, University of South-Eastern Norway, Kongsberg, Norway; 11 Department of Ophthalmology, Stavanger University Hospital, Stavanger, Norway

**Keywords:** Eyelid diseases, Eyelid diseases, Eye manifestations, Eye manifestations

## Abstract

Meibomian gland dysfunction (MGD) is the leading cause of dry eye and proposed treatments are based on disease severity. Our purpose was to establish reliable morphologic measurements of meibomian glands for evaluating MGD severity. This retrospective, cross-sectional study included 100 MGD patients and 20 controls. The patients were classified into dry eye severity level (DESL) 1–4 based on symptoms and clinical parameters including tear-film breakup time, ocular staining and Schirmer I. The gland loss, length, thickness, density and distortion were analyzed. We compared the morphology between patients and controls; examined their correlations to meibum expressibility, quality, and DESL. Relative to controls, the gland thickness, density and distortion were elevated in patients (p < 0.001 for all tests). The area under the receiver operating characteristic curve was 0.98 (95% confidence interval [CI], 0.96–1.0) for gland loss, and 0.96 (CI 0.91–1.0) for gland distortion, with a cutoff value of six distorted glands yielding a sensitivity of 93% and specificity of 97% for MGD diagnosis. The gland distortion was negatively correlated to the meibum expressibility (r = −0.53; p < 0.001) and DESL (r = −0.22, p = 0.018). In conclusion, evaluation of meibomian gland loss and distortion are valuable complementary clinical parameters to assess MGD status.

## Introduction

Meibomian gland dysfunction (MGD) is the leading cause of evaporative dry eye disease (DED)^1^. MGD can be both an asymptomatic, subclinical condition and a symptomatic disease. The disease progression is accompanied by specific clinical signs such as meibomian gland (MG) atrophy, altered MG secretion, and changes in lid morphology^2^.

The pathogenesis of MGD is thought to be gland obstruction due to hyperkeratinization, which blocks meibum secretion and causes meibum accumulation within the ducts, resulting in gland dilatation^2^. Gland enlargement could also be a compensatory mechanism for insufficient meibum secretion, and therefore can be an early finding of MGD^3^. The atrophic degeneration is thought to be a secondary response to increased pressure within the gland and appears in the later stages of the disease^4–6^.

Various clinical tests have been established for evaluation of MG function and morphology. Meibum quality and expressibility assessment are widely used for evaluating MG function^2,7^, whereas meibography can be applied for direct observation of MG morphological structure. The gland loss assessed by meibography images is a useful index of MGD^8–10^. Atrophy appears to occur in the later stages of the disease in contrast to gland dilatation, which may represent an early stage of MGD. Some research groups have described other changes in MG morphology such as MG thickness and length in dry eye patients^11–14^. Despite interesting findings, the reliability of these morphologic features remains unexplored, and their clinical utility is limited. Given the lack of better diagnostic tools, quantifying meibum quality and expression are essential in MGD diagnostics and are key evaluations in MGD stage grading^15^. In contrast, the role of morphological evaluation is underappreciated and not included in MGD classification.

The purpose of the present study was to establish reliable morphologic measurements of the MG for evaluating MGD severity.

## Results

Nine patients were excluded due to unsatisfactory meibography images according to the exclusion criteria. In total, 200 eyes of 100 patients (73 women and 27 men; mean age: 47.5 ± 15 years, age range: 11–82 years) and 40 eyes of 20 healthy volunteers (11 women and 9 men; mean age: 31.7 ± 14 years, age range: 19–65 years) were included, and a total of 480 images were evaluated.

### Diagnostic ability of clinical parameters for MGD

**Figure 1:**
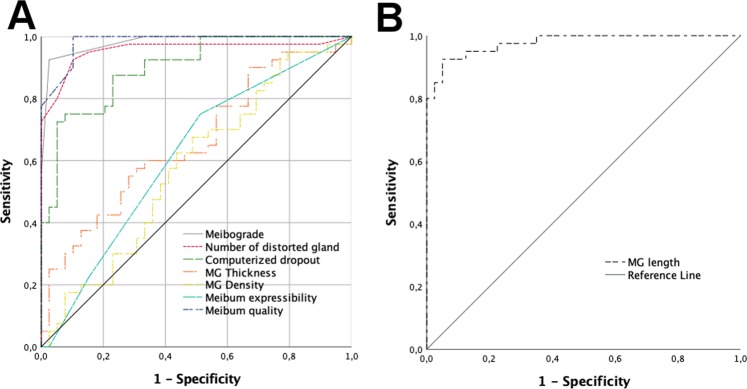
The receiver operator characteristic (ROC) curve for the detection of meibomian gland dysfunction (MGD) using morphologic measurements. (**A**) Represents the ROC curves for the detection of MGD by meibograde (AUC = 0.98), number of distorted glands (AUC = 0.96) computerized dropout (AUC = 0.69), MG thickness (AUC = 0.66), MG density (AUC = 0.58), meibum expressibility (AUC = 0.62) and quality (AUC = 0.98). (**B**) Illustrates the ROC curve for detection of MGD by MG length (AUC = 0,95). ROC; receiver operative characteristic curve, MGD; meibomian gland dysfunction, MG:meibomian gland, AUC; area under the ROC curve.

The meibograde, computerized dropout, number of distorted glands, MG thickness and density, and meibum quality were significantly elevated in the MGD patients compared to the healthy controls (Table 1). The patients had shorter MG length, while meibum expressibility was not significantly different. The ROC curves revealed the ability of morphological features to discriminate between MGD patients and healthy controls (Fig. 1). Meibum quality and meibograde both showed the greatest areas under the curve (AUC), both with a value of 0.98 (95% confidence interval [CI], 0.96–1.0). The optimal meibograde cut-off value was achieved using a total grade of both upper and lower eyelids for each side. A meibograde cut-off of 1.5 yielded 93% sensitivity and 97% specificity in discriminating MGD. The number of distorted glands had a high AUC (AUC = 0.96; 95% CI, 0.91–1.0), and the optimal cut-off value was six distorted glands, with 93% sensitivity and 90% specificity. The AUC for meibum expressibility was 0.62 (95% CI, 0.50–0.74).

**Table 1:** Comparison of morphologic and functional parameters between MGD patients and healthy controls.

	Patients (n = 100)	Controls (n = 20)	Mann-Whitney U P-value
Meibograde (score 0–6)	2.39 ± 1.63	0.35 ± 0.53	<0.001*
Computerized dropout (%)	35.6 ± 14.2	27.1 ± 13.3	0.022*
MG thickness (ImageJ pixels)	32.3 ± 10.6	20.9 ± 3.65	<0.001*
MG density (ImageJ pixels)	29.2 ± 8.9	20.4 ± 16.5	<0.001*
MG length (ImageJ pixels)	136.8 ± 52.9	298.5 ± 49.2	<0.001*
Number of distorted glands	6.8 ± 3.9	3.3 ± 2.1	<0.001*
Meibum quality (score 0–24)	9.8 ± 4.8	0.05 ± 0.22	<0.001*
Meibum expressibility (score 0–3)	1.0 ± 0.9	0.7 ± 0.8	0.387

MG = meibomian gland.

^*^Indicates significance after adjusting for the influence of age using a general linear model.

### Correlation between meibomian gland morphology and other clinical findings in MGD

Table 2 shows the correlation between MG morphology, DESL, and meibum expressibility and quality. In MGD patients, DESLcorrelated weakly with MG loss assessed by meibograde (r = 0.17, P = 0.006) and meibum expressibility (r = 0.21, P = 0.004). DESL was inversely correlated to MG length (r = −0.13, P < 0.001) and number of distorted glands (r = −0.22, P = 0.018). The number of distorted glands was further associated with meibograde (r = −0.60, P < 0.001). The MG loss by meibograde and computerized assessment were both correlated to meibum expressibility (r = 0.53; P < 0.001 and r = 0.43; P < 0.001, respectively), while number of distorted glands, MG thickness, density and length were inversely correlated to meibum expressibility (r = −0.53; P < 0.001, r = −0.27; P < 0.05, r = −0.53; P < 0.001, and r = −0.47; P < 0.001, respectively). Furthermore, the number of distorted glands for each meibum expressibility score was 7.1 ± 3.9, 6.5 ± 4.9, 5.1 ± 3.3, 2.5 ± 3.7 for score of 0, 1, 2, and 3, respectively (P < 0.001 for Kruskal-Wallis test). The number of distorted glands for each meibograde was 5.9 ± 3.6, 7.2 ± 4.2, 7.8 ± 3.2, 6.0 ± 3.6, 5.2 ± 4.0, 2.2 ± 2.8, 1.9 ± 2.6 for grade of 0 to 6 in an increasing order (P < 0.001 for Kruskal-Wallis test). The gland distortion was weakly associated with DESL, meibograde and meibum expressibility. The number of distorted glands was reduced with increasing meibum expressibility and meibograde.

**Table 2:** Pearson correlation between morphologic parameters, DESL, and meibum expressibility and quality.

	DESL (score 0–4)	Meibum expressibility (score 0–3)	Meibum quality (score 0–24)	No. of distorted glands
Meibograde (score 0–6)	0.17*	0.53^**^	0.18	−0.60^**^
Computerized dropout (%)	0.11	0.43^**^	0.12	−0.66^**^
MG thickness (ImageJ pixels)	−0.15^**^	−0.53^**^	0.03	−0.62
MG density (ImageJ pixels)	−0.06^**^	−0.27^*^	−0.04	0.35^**^
MG length (ImageJ pixels)	−0.13^**^	−0.53^* *^	−0.11	0.62^**^
Number of distorted glands	−0.22*	−0.47^* *^	−0.10	
Meibum quality (score 0–24)	0.06	−0.068		−0.24
Meibum expressibility(score 0–3)	0.21*			−0.47^**^

MG = meibomian gland.

^*^P < 0.05.

^**^P < 0.001.

### Reliability of measurements of meibomian gland morphology

Cohen’s kappa and the ICC were calculated to assess the reliability of the morphological quantifications. Cohen’s kappa values were 0.84 and 0.81 for intraobserver and interobserver agreements, respectively, in subjective meibograde. Similarly, the ICC values were between 0.81 and 0.89 in computerized MG dropout. Furthermore, the observers’ ability to repeat and reproduce morphological quantifications of MG length, thickness, and density and number of distorted glands yielded a range of ICC values of 0.56–0.94 (Table 3), referred with agreement^16–18^. Both subjective meibograde and computerized analysis of MG morphology demonstrated moderate to strong intra- and interobserver agreement.

**Table 3:** Kappa values and ICC for intra- and interobserver variability in evaluating MG morphology.

	*Cohen’s kappa (95% CI)*	*ICC (95% CI)*				
	Meibograde	Computerized dropout	MG thickness	MG density	MG length	Number of distorted MGs
Intraobserver	0.84 (0.80–0.88)	0.89 (0.84–0.92)	0.84 (0.74–0.90)	0.82 (0.73–0.88)	0.94 (0.91–0.96)	0.87 (0.79–0.92)
Interobserver	0.81 (0.76–0.86)	0.81 (0.75–0.86)	0.65 (0.14–0.83)	0.62 (0.45–0.74)	0.90 (0.56–0.96)	0.53 (0.08–0.75)

MG = meibomian gland.

Kappa values of <0.01, 0.01–0.20, 0.21–0.40, 0.41–0.60, 0.61–0.80, and 0.81–1.00 correspond to poor, slight, fair, moderate, substantial, and almost perfect agreement, respectively^16^.

ICC of <0.5, 0.5–0.75, 0.75–0.90, and 0.90–1.00 correspond to poor, moderate, good, and excellent reliability, respectively^18^.

## Discussion

The heterogeneous presentation of MGD complicates its detection and monitoring. Various treatments have been proposed based on MGD severity^15^, but the lack of a universally accepted staging system of clinical severity renders it problematic. The Tear Film & Ocular Surface Society (TFOS) attempted to establish an MGD grading system with focus on a few limited clinical findings, including altered meibum expression, meibum quality, and ocular staining^15^. Such a grading system underestimates the significance of MG anatomical changes in progressive MGD.

In the present study, we investigated multiple morphologic characteristics of MGs as assessed by meibography and examined their clinical application for evaluating MGD severity. We found that the meibograde, gland distortion, and MG length had excellent ability to discriminate between MGD patients and healthy subjects. Both meibograde and gland distortion were weakly correlated to DESL, meibum expressibility, and meibum quality suggests the necessity of MG morphology analysis in MGD development. Moreover, both subjective meibograde and computerized quantification of MG loss showed moderate to strong interobserver agreement indicating a great reliability for both analysis methods. The gland distortion is an early pathogenic finding and associated with progressive loss of MGs indicating severe MGD.

We found that a cut-off value of six distorted glands was sensitive and specific for diagnosing MGD. It was also observed that meibum expressibility decreases with progressive reduction in the number of distorted glands. Moreover, the MGD patients with worst meibum expressibility (score of 3) had the lowest number of distorted glands. Similarly, the lowest number of distorted glands was found in patients with highest meibograde (grade of 6). These findings suggest that MG torsion is, to a certain extent, pathogenic in early-stage MGD, and this particular structural change of MGs disappears with disease progression as MGs start to drop out.

The underlying mechanism of distorted MG development is unknown. The distorted MGs have also been observed in patients with allergic conjunctivitis^19^, and the duct distortion might represent an inflammatory process in early phase of MGD. However, the status of allergic conjunctivitis in MGD patients was not evaluated, and could therefore, be a confounding factor that contributes to observation of distorted glands^19^. Moreover, we observed that the decreased meibum secretion was also related to the reduced number of distorted glands. Our results indicate that finding of fewer distorted glands with increasing meibograde is associated with MGD development.

In addition to the number of distorted glands, we also found that the subjective meibograde and gland length were effective discriminators of MGD. In line with previous reports^2,20^, MGD patients had significantly higher MG dropout than the healthy controls. For this study, we used a modified four-point grading scale based on previously suggested cut-off values for MG dropout for discriminating between dry and normal eyes^21,22^. The results demonstrated a high efficacy of the meibograde for discrimination between MGD and healthy controls. Furthermore, a higher meibograde was associated with increased score of both meibum expressibility and quality. Taken together, our findings confirm that quantitative assessment of gland dropout is a sensitive and specific indicator of MGD development and progression.

Herein, we observed that MG length, thickness, and density were all weakly correlated with the meibum expressibility score, but not related to the quality of expressed meibum. These morphological changes may affect the secretion ability of a gland, but do not seem to affect the macroscopic quality of the secreted meibum. Our findings both agree with^11^ and contradict^12^ previous findings, complicating consistent interpretation of the results. It is, however, surprising that increased gland thickness was not related to altered meibum quality, as it has been hypothesized that gland obstruction and dilatation are partly driven by increased meibum viscosity^2^. On the other hand, it is impossible to evaluate meibum quality if a gland is completely obstructed and does not secrete meibum at all.

The tests of meibum expressibility and quality are considered a surrogate measure of MG function^2,7^, and our findings suggest that meibum quality is a sensitive and specific test for MGD. The diagnostic efficacy of meibum quality might be overestimated due to that MGD classification was based on meibum quality and expressibility. Nevertheless, the efficacy of meibum expressibility was poor, and may result from only assessing limited number of centrally located glands. It is known that the variable secretory activity of individual glands depending on their location along the eyelid^23^. In healthy subjects, the nasal MGs tend to produce more meibum^24^ and are more active even after considerable MG loss^25^. Thus, both nasal and temporal regions of the eyelid should be examined in future studies.

The reliability of clinical parameters is an important attribute of a consistent classification of MGD severity. In the present study, there was moderate to strong agreement among three observers regarding the quantification of morphologic features. Consistency was lowest for MG thickness and density. A possible explanation is interobserver disagreement in selecting the three most representative MGs, which will always be an issue in cases where all MGs are not evaluated. The variability between observers was, however, mitigated by using the average of three independent observers. These findings indicate that the clinical morphology parameters that are repeatable in a consistent manner should be chosen for evaluation of MGD and its severity. This approach may be useful in clinical practice, allowing investigators to standardize the quantification of morphologic features and to compare results obtained at different locations.

There are some limitations to the present study. The estimates on efficacy of meibum expressibility and quality score are subjected to the selection bias. The initial MGD diagnosis in current study was based on altered meibum expressibility or quality (score >1), and might consequently resulted in an overestimation of the diagnostic efficacy of those two tests. Second, some of the morphologic features were evaluated on the upper eyelids only. There are anatomical differences between the upper and lower eyelids; the lower eyelids have fewer glands^26^; while the lower eyelids have greater gland thickness, the gland length is shorter^11,21^. Future studies should include evaluation of the lower eyelids despite the strong correlation between the upper and lower eyelids^11,21^. Third, only the three most prominent glands were chosen for quantifying MG length, thickness, and density. There could potentially be interobserver disagreement in selecting the most representative glands. Despite the strong interobserver agreement in this study, a possible approach in future studies might be to investigate only a part of the eyelid, preferably the third part of the eyelid corresponding to the site where meibum expressibility and quality are tested.

Moreover, the observed morphological changes in patients could also be a result of confounding variables, including allergic conjunctivitis mentioned earlier and contact lens wear which has been reported to be associated with loss of MGs^27^. However, the potential for confounding factors was reduced by randomization of the group sample of patients and controls. Furthermore, the use of case-control in a study of diagnostic test may lead to inflated estimates of diagnostic accuracy compared to using a series of consecutive patients. Of note, none of the volunteers had any symptoms of ocular discomfort, which reduces the likelihood of additional conditions that potentially could generate false-positive results. Lastly, prospective studies are needed to confirm the utility of meiboman gland distortion cutoff as a diagnsotic parameter for MGD

In conclusion, structural MG changes are closely associated with MGD progression. More specifically, gland distortion, has a comparable diagnostic capability as MG loss and MG quality, and is therefore strongly affected by the pathological processes of MGD. Moreover, gland torsion is a pathogenic finding in the early stage, and associated with progressive loss of MGs in advanced stage MGD. Investigation of MG loss by meibograde and meibography visualization of the number of distorted glands are valuable complementary clinical parameters in assessing MGD status, and can be used for staging MGD severity.

## Materials and Methods

### Study subjects

One hundred and nine MGD patients and twenty healthy volunteers of mainly Caucasian ethnicity were evaluated in this retrospective, cross sectional, case-control study. MGD patients were selected from the patient pool from the Norwegian Dry Eye Clinic by a simple random sampling method. Results of a set of standardized clinical examinations including Ocular Surface Disease Index (OSDI) questionnaire, tear-film break-up time (TFBUT), Schirmer I test, ocular staining, meibum expresibility and quality, and meibographic imaging at their initial presentation to the clinic were analyzed.

The assessment of MGD is made after diagnosing DED, which was based upon symptom assessment and clinical tests as TFBUT, Schirmer I test and ocular surface staining^2^. Subjects with (1) score >1 for either meibum quality or expressibility or (2) score = 1 for both meibum expressibility and meibum quality, and over 20 years old were classified as MGD patients^2^. The patients were further evaluated with regard to the dry eye severity level (DESL) and scored with 1–4 according to the guidelines proposed by the 2007 International Dry Eye Workshop^28^. Briefly, DESL score was given based on a combination of severity of ocular symptoms and clinical ocular surface parameters, including TFBUT, ocular staining and Schirmer I (Table 4).

**Table 4:** Dry eye severity grading scheme.

Dry Eye Severity Level	1	2	3	4*
Discomfort, severity and frequency	Mild and/or episodic	Moderate episodic or chronic	Severe frequent or constant	Severe and/or disabling and constant
Conjunctival staining	None to mild	Variable	Moderate to marked	Marked
Corneal staining (severity/location)	None to mild	Variable	Marked central	Severe punctate erosions
TFBUT (sec)	Variable	≤10	≤5	Immediate
Schirmer I score (mm/5 min)	Variable	≤10	≤5	≤2

^*^Must have signs AND symptoms. TFBUT = tear-film breakup time.

Twenty healthy volunteers without any systemic diseases, pre-existing ocular conditions or dry eye symptoms were further recruited as a control group for this study. For control group, the clinical tests including TFBUT, Schirmer I, meibum expressibility, meibum quality were performed, and meibography images were also obtained.

The study was conducted in accordance with the Declaration of Helsinki. The Regional Committee for Medical & Health Research Ethics, Section C, South East Norway (REC) reviewed the use of the data in this study. REC found the research project “Evaluation of data from the Norwegian Dry Eye Clinic” to be outside the remit of the Act on Medical and Health Research (2008) and, therefore, could be implemented without specific approval. Written informed consent was obtained from all participants’ prior data collection.

### Morphology analysis

**Figure 2:**
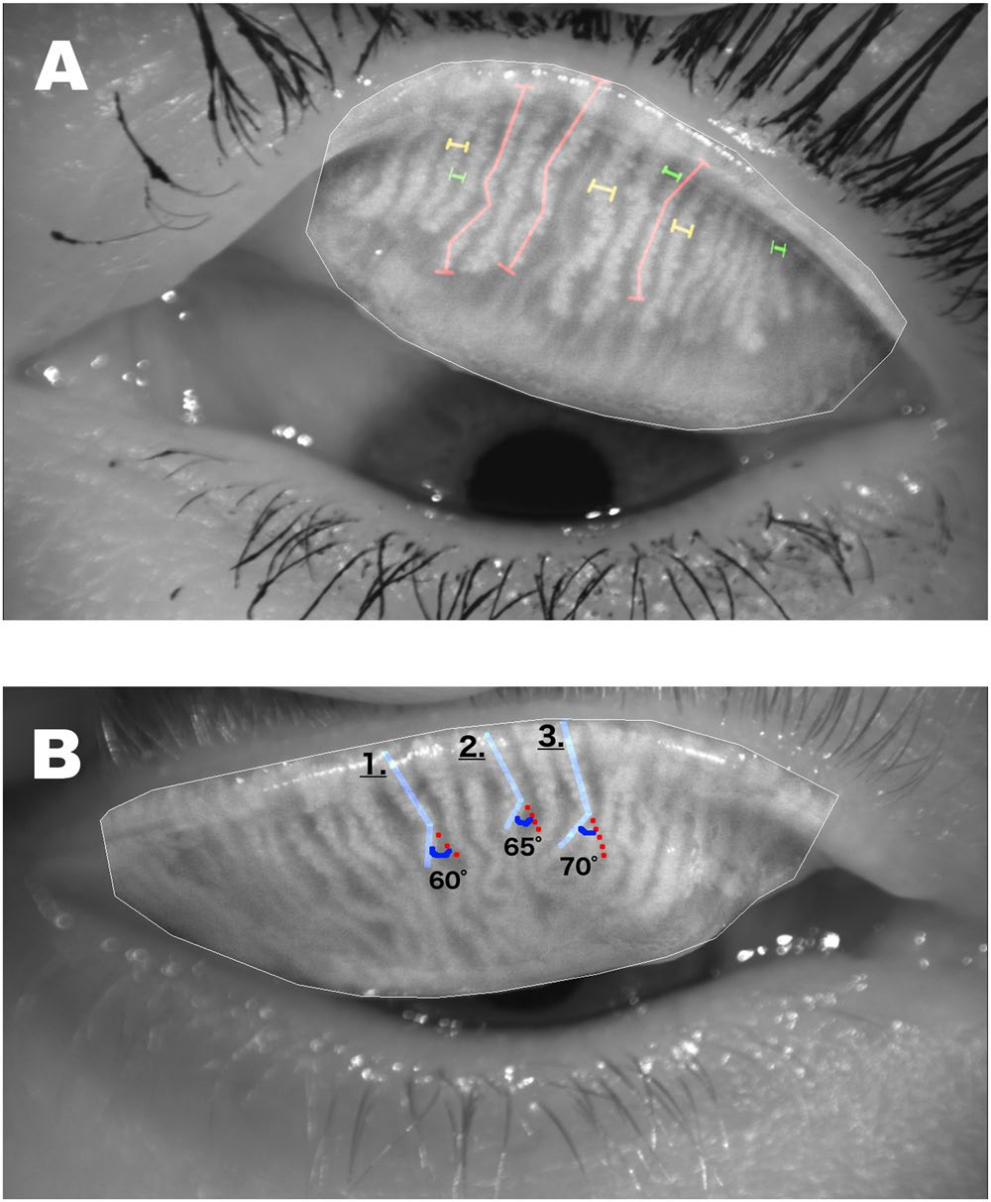
The number of distorted glands in the upper eyelids. (**A**) A total of 7 distorted glands were found in eyelid with meibograde 0. (**B**) The eyelid with meibograde of 2 had four distorted glands.

**Figure 3:**
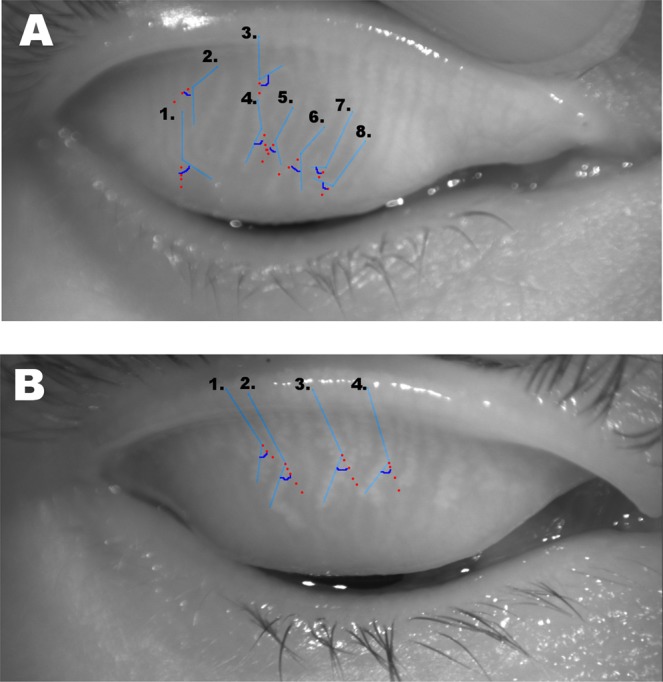
Computerized morphological measurements performed in the upper eyelid. (**A**) Three different measurements of MG length (red lines), thickness (yellow bars), and density (gap between two adjacent MGs indicated with green bars). (**B**) Calculation of the angle for counting the number of distorted glands in the upper eyelid.

The morphology was evaluated by analyzing meibography images obtained with the non-contact infrared meibography system OCULUS Keratograph 5 (OCULUS, Wetzlar, Germany). Images were excluded based on the following criteria: 1) interrupted complete assessment of the eyelid; 2) inadequate exposure of the tarsal area; 3) strong reflection of illumination; or 4) lack of focus of the image. MG loss in each eyelid was evaluated subjectively using a four-point grading scale (meibograde) of 0–3 as described in our previous work^22^: grade 0: 0–25% loss; grade 1: 26–50% loss; grade 2: 51–75% loss; and grade 3: >75% loss. The grades for both the upper and lower eyelids were summed to yield a total grade from 0 to 6 for each eye. MG dropout was also analyzed using computer and ImageJ software. Both MG loss and total tarsal area were measured as described by Pult *et al*.^21^, and the ratio was presented as the MG dropout percentage (0–100%). Further computerized analyses of additional morphologic characteristics were performed on the upper eyelids only. For MG thickness and length measurements, three glands mainly in central region, with length and thickness in close approximation to majority of the glands were subjectively chosen as most representative glands and analyzed. MG area density was assessed by measuring the interglandular space between two adjacent MGs at three different sites on the eyelid (Fig. 2A)^22^. A larger interglandular space value indicated lower density. For measurement of MG length, a continuous line following the path of the gland and covering the entire visible length of a gland was drawn and measured. To depict the MG thickness a continuous horizontal line covering the gland horizontally was drawn and measured. To measure the interglandular space, a continuous horizontal line was drawn between the outer borders of two adjacent glands and measured. Lastly, the number of distorted MGs (with torsion >45°) in upper eyelid was counted (Fig. 2B) and represents level of gland distortion for each eye (Fig. 3).

Three experienced observers analyzed the meibography images to assess the interobserver reliability. The observers repeated their analyses at a 2-week interval to evaluate intraobserver agreement. The observers were masked for the diagnosis, from other observers, and their own previous analyses.

### Clinical tests of meibomian gland function

All patients first completed a symptom questionnaire to obtain an OSDI score between 0 (no symptoms) and 100. Five MGs in the central area of the lower eyelids were tested for their ability to express meibum. The ability of these glands to secrete meibum was graded 0–3 based on the number of expressible glands as described by Pflugfelder *et al*.^29^: 0 = all glands expressible; 1 = 3–4 glands expressible; 2 = 1–2 glands expressible; and 3 = no glands expressible. Meibum quality was assessed on the central 8 MGs in the lower eyelids, and rated on a 0–3 scale: 0 = clear fluid; 1 = cloudy fluid; 2 = cloudy, particulate fluid; and 3 = inspissated, toothpaste-like meibum^30^. The score for each expressed gland was summed to yield a composite score^2^. The Schirmer I test was performed without anesthesia by inserting the test strip in the lateral third of the lower eyelid for 5 minutes^4^. TFBUT for each eye was measured 30 seconds after instillation of 5 µl 2% fluorescein to the conjunctival sac. Ocular surface fluorescein staining was analyzed in similar fashion and graded using the Oxford grading system^4,31^.

### Statistical analysis

Data were analyzed with SPSS (v24.0). Cohen’s kappa values were calculated to evaluate the observers’ agreement of the subjective meibograde, and intraclass correlation coefficient (ICC) was evaluated for the consistency of computerized measurements of morphology. A principal component analysis (PCA) was performed to take into account and summarize the inter-eye correlation. PCA is a statistical data reduction technique used to explore the directions of maximal collinearity among a group of variables^32,33^. In this study, the result of the individual parameter from both eyes of each subject was optimally weighted using PCA loadings, so that a single factor score characterizing each subject could be obtained and used for further statistical analysis^34^. Relationships between morphological features and MG function were determined by Pearson correlation. The patients and healthy subjects were compared using the Mann-Whitney U statistics and Kruskal-Wallis with Dunn’s post-hoc test. The influence of age in between-group comparisons was adjusted using a general linear model. A receiver operator characteristics (ROC) curve was generated to investigate the clinical application and optimal cut-off values of morphologic measures in MGD diagnostics. P < 0.05 was considered statistically significant.

## Data Availability

The datasets generated during and analyzed during the current study are available from the corresponding author on request.
